# Mitigation of Expression of Virulence Genes in *Legionella pneumophila* Internalized in the Free-Living Amoeba *Willaertia magna* C2c Maky

**DOI:** 10.3390/pathogens9060447

**Published:** 2020-06-05

**Authors:** Rayane Mouh Mameri, Jacques Bodennec, Laurent Bezin, Sandrine Demanèche

**Affiliations:** 1 R&D Department, Amoéba, 69680 Chassieu, France; mameri77@yahoo.fr; 2 Lyon Neuroscience Research Center CRNL UMR5292 U1028, University of Lyon, Univ Lyon 1, CNRS, Inserm, 69500 Bron, France; jacques.bodennec@univ-lyon1.fr (J.B.); laurent.bezin@univ-lyon1.fr (L.B.)

**Keywords:** free-living amoebae (FLA), *Legionella pneumophila*, virulence genes, *Willaertia magna* C2c Maky

## Abstract

*Legionella pneumophila* is a human pathogen responsible for a severe form of pneumonia named Legionnaire disease. Its natural habitat is aquatic environments, being in a free state or intracellular parasites of free-living amoebae, such as *Acanthamoeba castellanii*. This pathogen is able to replicate within some amoebae. *Willaertia magna* C2c Maky, a non-pathogenic amoeba, was previously demonstrated to resist to *L. pneumophila* and even to be able to eliminate the *L. pneumophila* strains Philadelphia, Lens, and Paris. Here, we studied the induction of seven virulence genes of three *L. pneumophila* strains (Paris, Philadelphia, and Lens) within *W. magna* C2c Maky in comparison within *A. castellanii* and with the gene expression level of *L. pneumophila* strains alone used as controls. We defined a gene expression-based virulence index to compare easily and without bias the transcript levels in different conditions and demonstrated that *W. magna* C2c Maky did not increase the virulence of *L. pneumophila* strains in contrast to *A. castellanii*. These results confirmed the non-permissiveness of *W. magna* C2c Maky toward *L. pneumophila* strains.

## 1. Introduction

The genus *Legionella* includes more than 60 species, but human infections that progress to a severe pneumonia, known as Legionnaires’ disease, are most often caused by *Legionella pneumophila* [1,2]. This Gram-negative bacterium especially affects immunocompromised individuals after inhalation of Legionella-contaminated aerosols. *L. pneumophila* invades and replicates within alveolar macrophages and epithelial cells of the lungs, inducing a severe respiratory infection [1,3].

*L. pneumophila* is ubiquitous in natural, artificial, and industrial aquatic environments and is mostly nested in intracellular hosts, such as free-living amoebae (FLA). In 1980, Rowbotham first demonstrated the intracellular multiplication of *L. pneumophila* within *Acanthamoeba* spp. and *Naegleria* spp. [4]. Following this study, several reports described the replication of *Legionella* isolates from clinical samples within protozoa isolated from the presumed source of infection. Intracellular growth within protozoa was shown to increase the ability of *L. pneumophila* to infect human monocytes and to resist to chemical disinfectants, biocides, and antibiotics [5,6,7]. Inhalation of legionellae packaged in amoebae is associated with the induction of more severe clinical cases of legionellosis. The speculated link between the homing of legionella in amoebae and the increased virulence of legionella is supported by the publication of a mouse model of co-inhalation of *L. pneumophila* and *Hartmannella vermiformis*. It was shown to significantly enhance the intrapulmonary growth of *L. pneumophila*, with a greater mortality than that observed from inhalation of legionellae alone [8]. The intra-amoebae growth was demonstrated to enhance the ability of *L. pneumophila* to infect epithelial cells (100- to 1000-fold), murine macrophages (10- to 100-fold), human monocytes (100- to 1000-fold), and *Acanthamoeba castellanii* (10- to 100-fold) [9]. Moreover, *L. pneumophila* grown in *A. castellanii* displays enhanced infection in monocytes compared to buffered charcoal yeast extract (BCYE)-grown bacteria [10]. Within FLA and human macrophages, *L. pneumophila* cells are able to reroute the phagosome thanks to a functional Dot/Icm type 4 secretion system (T4SS) and the approximately 300 proteins it secretes and to induce the biogenesis of a legionella-containing vacuole (LCV) [11,12]. Bacterial replication occurs in LCV, evades fusion with lysosomes, and associates intimately with the host endoplasmic reticulum (ER), inducing the lysis of the host cells. The induction of apoptosis in the host cell is induced by a type IV-like secretion machinery [13]. The internalization of *L. pneumophila* into host cells, such as FLA, promotes not only its ability to survive and multiply but also to acquire and increase its virulence [14]. The *L. pneumophila* Dot/Icm-secreted effector PlcC/CegC1 was demonstrated to promote virulence [15], and the translocated Dot/Icm type IVB secretion system effector SdhA was demonstrated to be of crucial importance in infection processes [16].

Genes of the T4SS system have also been identified, with several other genes, as being responsible for the increased virulence in *L. pneumophila* once internalized by FLA, such as *A. castellanii* and *Vermamoeba vermiformis* [17]. In two studies from NJ Ashbolt’s laboratory, about 30 transcripts of genes involved in bacterial metabolism, replication, and virulence have been investigated using reverse transcription quantitative polymerase chain reaction (RT-qPCR) in *L. pneumophila* Philadelphia after exposure to CuO nanoparticles (CuO-NPs) or synthetic gray water (Gw) for a period ranging from 3 to 48 h [18,19]. 

Here, we investigated the expression level by RT-qPCR of 7 genes (*htpX*, *icmE*, *lirR*, *ccmF*, *gacA*, *tatB*, and *lvrE*) that we have shown to be expressed by all three reference strains, *L. pneumophila* Paris, Philadelphia, and Lens, after co-incubation with two FLA: *A. castellanii* known to be permissive to the legionella multiplication [10], and *Willaertia magna* C2c Maky considered as non-permissive to the legionella multiplication [20]. We also evaluated transcript levels of these genes in *L. pneumophila* cultivated alone and harvested at the end of the growth exponential phase corresponding to the end of the replicative phase and the beginning of the virulence phase. The aim of this work was to evaluate the evolution of virulence of *L. pneumophila* strains after internalization into a non-permissive amoeba, such as *W. magna* C2c Maky. To facilitate the interpretation and comparison of gene expression, a GENE EXPRESSION-based index was developed.

## 2. Results

### 2.1. Virulence Gene Selection

Virulence genes of interest were selected based on previous studies [18,19] that investigated the potential increase in virulence gene expression in L. pneumophila Philadelphia exposed to CuO-NPs or synthetic Gw (Table S1). As our objective was to assess virulence gene expression in *L. pneumophila* internalized for three days in amoebae, we focused on genes whose expression was increased after 24–48 h of environmental exposure to CuO-NPs (*dotA*, *enhC*, *htpX*, *icmE*, and *pvcA*) or synthetic Gw (*lirR*, *ccmF*, *gacA*, *tatB*, *lvrB*, and *lvrE*). We excluded the genes *cegC1* and *sidF* displaying conflicting variations between the two environmental conditions (Table S1).

Among the 11 genes selected, we were not able to amplify *pvcA* cDNA in *L. pneumophila* Philadelphia using published primers [19]. We thus ran qPCR for the other 10 genes, using the primers listed in Table 1.

**Figure 1:**
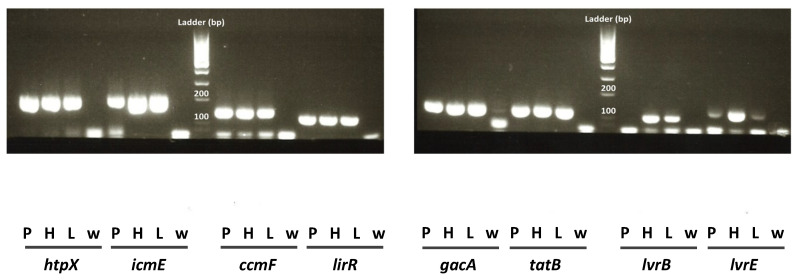
Amplicon size verification on 2% agarose gel for the three L. pneumophila strains. bp: base pair; P: strain Paris; H: strain Philadelphia; L: strain Lens; w: water. The ladder is the MassRuler Low Range DNA Ladder (Thermo Fisher Scientific, Lyon, France SM0383); the 100 and 200 bp bands are indicated.

As expected, these primers allowed high quality amplification of the targeted cDNAs in *L. pneumophila* Philadelphia cultured for three days in BCYE plates, with the exception of *dotA* and *enhC* cDNAs. Indeed, the amplification of these two amplicons did not reach satisfactory criteria for either the melt curve analysis or the agarose gel electrophoresis of the end products, for the three *L. pneumophila* strains. We thus excluded these two genes from the final analysis. The primers used for the eight remaining genes produced good amplifications, with end products of the expected size (Figure 1). However, the expression of *lvrB* in *L. pneumophila* Paris was not detected in all conditions tested. It is possible that the *lvrB* gene differs in *L. pneumophila* Paris from the two other strains, in regions recognized by the primers used. To avoid any bias when comparing the three strains, *lvrB* was not included in the final list of the seven selected transcripts.

### 2.2. Validation of rpsL as a Non-Acceptable Housekeeping Gene, in Which Transcript Level Should Be Stable between the Different Tested Conditions

While real-time PCR is a quantitative method with high sensitivity and great reproducibility, to measure targeted DNAs over a wide range of concentrations, the RT of biological sample RNAs is a reaction that is difficult to calibrate. To normalize differences in RT efficiency across samples, the transcript level of a gene, mostly a housekeeping gene, is used as an internal control, whose expression is supposed to be invariant between various treatments or conditions. The ribosomal gene *rpsL* is a housekeeping gene commonly used for phylogenic analysis of *Legionella* [21] and has been used to normalize the reverse transcription of RNA extracted from *L. pneumophila* exposed to Cuo-NPs nanoparticles or synthetic Gw [18,19]. An alternative way to normalize the RT reaction between the different samples is to use a known quantity of synthetic RNA, added directly to the reaction mix, thereby allowing the same number of copies of this synthetic RNA to be added across all samples. As an external control, we used the so-called synthetic non-homologous standard mRNA (SmRNA) [22], of which we ensured that the primers used for the amplification of its cDNA do not amplify DNA sequences resulting from the RT of the endogenous RNAs of *L. pneumophila*.

**Figure 2:**
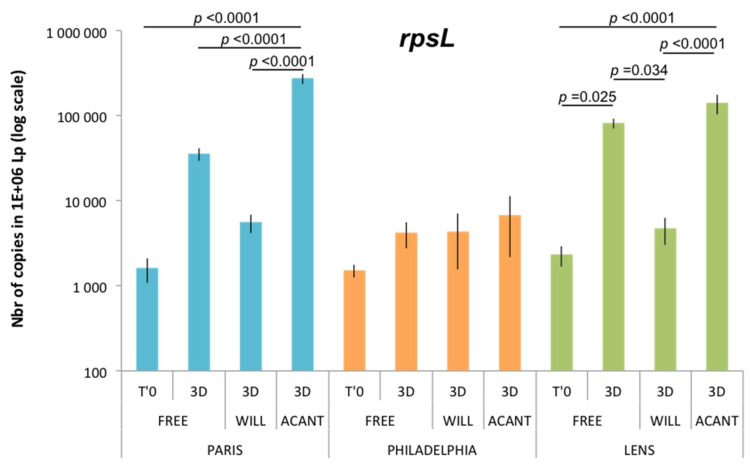
Level of *rpsL* transcript in the different conditions, expressed as the number of copies in 1 × 10^6^ *L. pneumophila* (Lp) ± SD and displayed using a log scale. Abbreviations: FREE: *L. pneumophila* strains alone; WILL: *L. pneumophila* strains cocultured with *W. magna* C2c Maky; ACANTH: *L. pneumophila* strains cocultured with *A. castellanii*; T′0: reference transcript level; 3D: transcript level after 3 days. ANOVA 2: Factor 1: “*L. pneumophila* strain”, *p* < 0.0001; Factor 2: “culture conditioned”, *p* < 0.0001; Interaction Factor 1 × Factor 2, *p* < 0.0001.

Thus, using SmRNA as an external control, the expression of *rpsL* could be examined by RT-qPCR like any other gene. Using a graphical representation with an ordinate axis on a linear scale, we show that there is a high variability in the *rpsL* mRNA level, in particular in *L. pneumophila* Paris and *L. pneumophila* Lens (Figure S1). In a logarithmic representation of the ordinate axis, one can note that the *rpsL* mRNA level was almost identical between the three *Legionella* strains under the T′0 control condition. In addition, the *rpsL* mRNA level in the *L. pneumophila* Philadelphia strain remained unchanged under the various conditions tested. On the other hand, the maintenance for three days of the incubation of the *L. pneumophila* Paris and *L. pneumophila* Lens strains in the liquid medium (3D-FREE) caused an increase in the *rpsL* mRNA level, which was significant compared to the T′0 control in *L. pneumophila* Lens. The internalization of *L. pneumophila* Paris and *L. pneumophila* Lens in *A. castellanii* was also followed by a strong and significant increase in the *rpsL* mRNA level, not only compared to the T′0 condition but also compared to the T3D-FREE condition (*A. castellanii* only). Finally, the internalization of the three strains in *W. magna* C2c Maky did not lead to a significant increase in the *rpsL* mRNA level (Figure 2).

Given that *rpsL* is not a gene whose expression is invariant under the conditions tested, we did not use it for the normalization of the RT reaction and instead used the SmRNA for all of the targeted genes.

### 2.3. Definition of a Gene Expression-Based Virulence Index

Studies aimed at investigating virulence gene expression usually analyze each gene separately, making it difficult to draw clear conclusions, especially when expression increases for some genes, decreases for others, and finally remains stable for the latest.

Since qPCR makes it possible to quantify cDNA copies in a sample, we eluded the above-mentioned issue by defining for each sample a virulence index, which is the sum of all virulence cDNAs quantified by qPCR.

However, in the calculation of the virulence index, we paid attention to avoid masking important variations for genes expressed at low levels in basal conditions by genes initially expressed at high levels. To this end, for each transcript, the cDNA copy number contained in a given sample has been expressed as a percentage of the averaged copy number measured in all samples. 

### 2.4. Comparison of the Virulence Index of three L. pneumophila Strains

**Figure 3:**
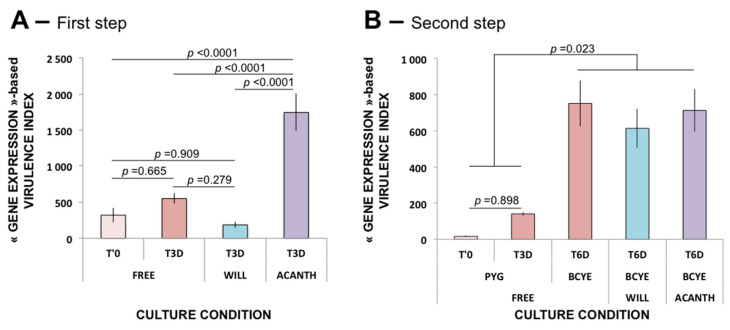
Measurement of the virulence index. (**A**) After 3 days in liquid medium; (**B**) after 3 days in liquid medium plus 3 days on buffered charcoal yeast extract (BCYE) plates. FREE: *L. pneumophila* strains alone; WILL: *L. pneumophila* strains cocultured with *W. magna* C2c Maky; ACANTH: *L. pneumophila* strains cocultured with *A. castellanii*; T′0: reference virulence index; T3D: virulence index after 3 days; T6D: virulence index after 6 days, 3 days in liquid medium, and 3 days on BCYE plates.

Firstly, we evaluated the “Gene Expression-based” virulence index of three strains of *L. pneumophila* after coincubation for three days within two amoebic species: *W. magna* C2c Maky and *A. castellanii*, (Figure 3A), based on the measurements of the transcript levels of the seven virulence genes selected: *ccmF* (Figure S2), *gacA* (Figure S3), *htpX* (Figure S4), *icmE* (Figure S5), *lirR* (Figure S6), *lvrE* (Figure S7), and *tatB* (Figure S8). These measures were performed after three days of coculture when the amount of intracellular *Legionella* was not significantly different between *W. magna* C2c Maky and *A. castellanii*.

Secondly, after Day 3, every condition (*L. pneumophila* alone, *L. pneumophila* strains co-incubated with *W. magna* C2c Maky, and *L. pneumophila* strains co-incubated with *A. castellanii*) was seeded on BCYE plates for three additional days (until Day 6) to remove all amoeba traces, because amoebae are not able to survive on a BCYE plate, and to evaluate the fate of the virulence of *L. pneumophila* after their release from amoebae. Once *L. pneumophila* had grown on the BCYE plates, bacteria were harvested, and the virulence index was evaluated (Figure 3B) as above, based on the measurements of the transcript levels of the seven virulence genes selected: *ccmF* (Figure S9A), *gacA* (Figure S9B), *htpX* (Figure S10A), *icmE* (Figure S10B), *lirR* (Figure S11A), *lvrE* (Figure S11B), and *tatB* (Figure S12). As expected, the transcript levels varied differently between the different genes selected, making it difficult to draw a clear conclusion (Figures S2–S12).

A virulence index was thus calculated for each condition, and an ANOVA 2 revealed for Steps 1 and 2 (Figure S13A,B) that the three *L. pneumophila* strains behaved in the same way under all tested conditions. Their results were then pooled and averaged (Figure 3).

ANOVA 2 also revealed a statistical difference between culture conditions (*p* < 0.0001), and post-hoc analysis (Tukey’s HSD test) showed a significant increase of the virulence genes after the internalization of *L. pneumophila* in *A. castellanii* compared to the controls containing *L. pneumophila* alone at Day 0 and Day 3 (Figure 3A). Conversely, a tendency (not significant) to decrease the level of virulence of the three *L. pneumophila* strains within *W. magna* C2c Maky was observed when compared to *L. pneumophila* alone (control) at Day 0 and Day 3 (Figure 3A). *L. pneumophila* strains internalized within *W. magna* C2c Maky exhibit a virulence index that is not statistically different from the controls at T’0 and T3D, demonstrating that *W. magna* C2c Maky did not increase the virulence of *L. pneumophila* strains in contrast to *A. castellanii*.

## 3. Discussion

These experiments brought evidence of a different behavior between *L. pneumophila* strains internalized by *W. magna* C2c Maky and strains internalized by *A. castellanii*. Indeed, the expression of virulence genes is reduced in *L. pneumophila* internalized into *W. magna* C2c Maky cells, while it is significantly increased in *L. pneumophila* internalized into *A. castellanii*.

These results confirm the non-permissiveness observed in *W. magna* C2c Maky towards *L. pneumophila*. Unlike *A. castellanii*, *W. magna* C2c Maky is able to internalize and digest *L. pneumophila* by phagocytosis [20,23]. These data suggest that *L. pneumophila* strains were unable to use their T4SS system to deflect the cellular machinery of *W. magna* C2c Maky. As a consequence, the phago-lysosomal fusion could happen and LCV could not be created, leading to the intracellular destruction of bacteria.

Considering FLA known to be permissive to *L. pneumophila* multiplication, such as *A. castellanii*, Buse et al. reported that the Dot/Icm T4SS system was responsible for the translocation of a large number of Legionella effectors in the host cells to promote infectivity [18]. These translocated effector genes are located in a hypervariable region of the *L. pneumophila* genome, and the plasminogen activator homologue of *L. pneumophila*, was strongly involved in *A. castellanii* intracellular growth [24,25]. The virulence associated genes *lvrB* and *lvrE* were also shown to be involved in the T4SS [21,26]. Another effector of the Dot/Icm substrate, SidF, was shown to be involved in the inhibition of programmed cell death in the host and anchoring of binding effectors to bacterial phagosomes [27,28]. However, Legionella *vir* homologues were shown to be not required for intracellular replication in amoeba or macrophage [21].

Surprisingly, *L. pneumophila* strains cultivated alone in liquid medium for three days and then incubated for three additional days on BCYE plates exhibit a great increase of the virulence index (Figure 3B), demonstrating an induction of the expression of virulence genes. ANOVA 2 revealed a statistical difference between culture conditions (*p* < 0.0001). Post-hoc analysis (Tukey’s HSD test) showed a tendency of the virulence index to increase from Day 0 to Day 3 under the control conditions (not significant, *p* = 0.898) and a clear statistical difference from other conditions (Figure 3B), i.e., the control at Day 6, and the *L. pneumophila* strains internalized within *W. magna* C2c Maky and *A. castellanii* at Day 6 (*p* = 0.023). No statistical difference was observed at Day 6 between the control condition and the *L. pneumophila* strains co-cultivated with both amoebae. It seems that cultivation on BCYE plates erased the virulence index acquired during internalization in amoebae to confer a virulence index specific to BCYE culturing. Bacteria cultivated on BCYE plates were shown to be virulent, even if some isolates transiently lost their flagella. Indeed, some isolates characterized as aflagellate when harvested from BCYE agar were shown to be able to multiply in amoebae, and flagella were subsequently detectable by immunologic methods [29]. Moreover, Nowicki et al. have concluded that infection with aerosols of *L. pneumophila* coming from cultures on BCYE plates causes mortality in guinea pigs, showing that BCYE-cultivated *Legionella* are indeed virulent [30]. Consequently, the virulence index can only be assessed in conditions of liquid culture without sub-culturing on BCYE plates.

To conclude, we developed a tool that can be named the “GENE EXPRESSION-based Virulence Index” based on the quantitative measurement of cDNAs of 7 virulence genes (*ccmF*, *gacA*, *htpX*, *icmE*, *lirR*, *lvrE*, and *tatB*) using a calibrated RT and real-time PCR. Virulence genes were expressed at low levels in the three strains of *L. pneumophila* in the absence of amoebae, making it possible to determine a “GENE EXPRESSION-based Virulence Index” both at T0 and after 3 days in a liquid medium.

*L. pneumophila* strains followed a similar trend, since the “GENE EXPRESSION-based Virulence Index” between the three strains was not statistically different. After internalization in *W. magna* C2c Maky, there was no increase in the “GENE EXPRESSION-based Virulence Index” compared to the control conditions, since no statistical difference between the index determined after internalization in *W. magna* C2c Maky and the index determined in control *L. pneumophila* was observed for the three *L. pneumophila* strains. A huge and significant increase in “GENE EXPRESSION-based Virulence Index” was observed in the three tested *L. pneumophila* strains internalized in *A. castellanii* compared to *W. magna* C2c Maky, with a fold difference between the two amoebae reaching 9.4 (Virulence Index was 185 ± 44 in WILL condition and 1745 ± 257 in ACANTH condition). These data confirmed that *W. magna* C2c Maky cells resist *Legionella* strains, are able to phagocyte *Legionella* strains, and do not increase their virulence after internalization.

This index synthesized the expression of all the genes in one data for each condition, rendering the comparison easier. Moreover, a particular attention was brought to the genes that were expressed at low level by expressing the cDNA copy number contained in a given sample in percent of the averaged copy number measured in all samples. This GENE EXPRESSION-based Virulence Index could be used in many studies that focus on the variation of gene expression in a given function (here virulence, elsewhere inflammation, development, cell death, etc.) where many transcripts are analyzed. When different genes associated with the same function are compiled or integrated in the form of an index, it makes it easier to conclude about the overall evolution of these genes, a conclusion often made difficult when the individual variations do not always point in the same direction.

## 4. Materials and Methods

### 4.1. L. pneumophila Cultures

The three strains of *L. pneumophila* serogroup 1, Lens CIP 108 286, Paris CIP 107 629T, and Philadelphia ATCC 33152, were cultured at 36 °C for 3 days on buffered charcoal yeast extract (BCYE) agar plates (Thermo Fisher Scientific, Dardilly, France) before coculture experiments. Quantification of *L. pneumophila* was performed via Real-time qPCR using Biorad kits (Biorad, Hercules, CA, USA). Briefly, aliquots of cell suspension were sampled, and genomic DNA was extracted using the AquadienTM DNA extraction kit. *L. pneumophila* was quantified using the iQ-CheckTM L. pneumophila Real-time qPCR kit and a CFX96 Biorad thermocycler. The data were analyzed using the Bio-Rad CFX Manager Industrial Diagnostic Edition software (version 2.2). The whole procedure was designed for quantification of *L. pneumophila*, including intra-amoebic bacteria, and has been validated by AFNOR (Agence Française de Normalisation, the French standardization authority).

### 4.2. FLA Culture

The amoebae used in this study were *W. magna* C2c Maky ATCC PTA-7824 and *A. castellanii* ATCC 30010. Amoebae were grown at 30 °C for 3 days using adhesion culture on CF4 with serum casein glucose yeast extract medium (SCGYEM) [31]. Amoeba cells were maintained in the exponential growth phase by subculturing every 3 days. Quantification of amoeba populations was performed using 0.1 mL of each aliquot utilizing a hemocytometer cell counting chamber method with Trypan blue.

### 4.3. Coculture of L. pneumophila with Amoebae

Tubes containing 5 mL of Peptone Yeast Extract Glucose Broth (PYG) [32] were seeded with 5 × 10^5^ *W. magna* C2c Maky or *A. castellanii* cells. At Day 0, the different strains of *L. pneumophila*, grown on BCYE plates, were suspended in sterile distilled water at 2.5 × 10^8^ cells/mL, and inoculated into the amoebic cultures at a multiplicity of infection (MOI) of 50. Low-speed centrifugation (30 min at 1000× *g*) was used to initiate physical interaction between bacteria and amoebae, and then incubated at 36 °C for 1 h. To eliminate extracellular legionella, cocultures were treated for 2 h at 30 °C with 0.5 mg/mL of Penicillin and Streptomycin. Cells were then washed twice by, first, centrifuging assay tubes for 10 min at 1000× *g*, second, removing the supernatant, and, finally, adding 5 mL of fresh PYG medium preheated at room temperature. After the second wash, cocultures were incubated at 30 °C in PYG medium for 3 days (Figure S13A).

In a second step, coculture solutions were submitted to a mechanical cell lysis using a FastPrep^®^-24 instrument (MP Biomedicals, Illkirch-Graffenstaden, France) for 2 × 30 s at a speed of 5.0 in order to release legionella from the amoebae. Afterward, 100 µL of each treated coculture were deposited onto BCYE plates and incubated for 3 days at 36 °C (Figure S13B).

Control conditions consisted in legionella cultured in PYG medium in the absence of amoebae (FREE condition) for 3 days; for the second step, 100 µL of the latter culture were deposited onto BCYE plates for 3 additional days (Day 6), as described above.

### 4.4. Preservation of RNA Samples

Free legionella and cocultures (amoebae with internalized legionella) in liquid medium, or legionella colonies on BCYE plates were collected, rinsed in sterile osmosed water, and centrifuged for 5 min at 4 °C at 6000× *g*. The TRIzol^®^ Max™ Bacterial RNA Isolation Kit (Thermo Fisher Scientific, Lyon, France) was used to improve the isolation of intact total RNA. The kit utilizes both the Max™ Bacterial Enhancement Reagent and TRIzol^®^ Reagent to inactivate endogenous RNases and promote protein denaturing, improving RNA quality and integrity. After removal of the supernatant, 200 µL of pre-heated (95 °C) Max Bacterial Enhancement Reagent buffer was added on the pelleted cells and incubated for 5 min at 95 °C. Afterward, 1 mL of TRIzol Reagent was added, and the tubes were frozen in dry ice before being stored at −80 °C.

### 4.5. Total RNA Extraction

Biological material in TRIzol^®^ was used for total RNA extraction according to the manufacturer’s instruction and MIQE guidelines [33]. Total nucleic acid extracted was treated with Turbo DNA-free DNAse (Thermo Fisher Scientific, Lyon, France) as recommended by the manufacturer, to remove genomic DNA. Total RNA was then transferred into a nuclease-free tube and stored at −80 °C after the concentration was measured using a Biodrop spectrophotometer (Biodrop, Cambridge, UK).

### 4.6. Calibrated Reverse Transcription (cRT)

Briefly, 180 ng of purified total RNAs were used for cRT using random hexamers and Multiscribe™ reverse trancriptase in the presence of RNAse inhibitor (Thermo Fisher Scientific). A synthetic external and non-homologous Standard RNA (SmRNA) was used to control the quality of the RT and to normalize the reverse transcription of mRNAs of biological samples [34]. At the end of the RT, cDNAs were stored at −20 °C until further use. It should be noted that the Multiscribe^®^ reverse transcriptase was not added in the RT master mix as recommended by the manufacturer. Indeed, thanks to the data obtained for the SmRNA, we discerned that the enzyme was rapidly altered in the master mix, thus significantly affecting the efficiency of reverse transcription across samples. This problem was resolved by preparing a master mix without the enzyme and then by adding the enzyme directly in each individual reaction tube.

### 4.7. Quantitative PCR (qPCR)

qPCR was performed using a Rotorgene Q (Qiagen, Courtaboeuf, France) and the Rotor-Gene SYBR Green PCR kit (Qiagen). All qPCR assays were run under the following conditions: 95 °C for 5 min, followed by 40 cycles of 5 s at 95 °C and 20–45 s (depending on the size of the amplicons) at 60 °C. The melting curve analysis was performed to evaluate the specificity of the DNA amplified during the PCR. The cycle of quantification Cq was determined at the intersection between the threshold line and the amplification curves when data were displayed as semi-logarithmic representation of the accumulated fluorescence versus cycle number. Cq was then transformed into a number of cDNA copies, according to standard curves composed of standards with known copies of cDNAs (1 to 10^8^ copies). These standard curves reliably indicated the method’s detection sensitivity to targeted cDNAs (1 copy detected in 25% of cases) and the linear range of the quantification (from 10 to 10^8^ copies). Primer sequences, the slope of the standard curve established between the Cq and the initial cDNA concentration, as well as the efficiency of the qPCR of each targeted cDNAs, are presented in Table 1.

### 4.8. Calculation of the Virulence Index

As mentioned above (Section 2.3), the cDNA copy number contained in a given sample has been expressed in percent of the averaged copy number measured in all samples: 36 samples in each series of Task-1 (Lp Paris + Lp Philadelphia + Lp Lens) and 30 samples in Task-2 (Lp Paris + Lp Lens). Thus, at the end of each experiment series, and for each gene, the averaged “balanced” cDNA copy number calculated from all samples equaled 100. Finally, for a given sample, Gene expression-based Virulence Index was calculated as follows:

$$\mathrm{Virulence}-\mathrm{Index}\;\mathrm{for}\;\mathrm{sample}\;A\;=\overset{7}{\underset{n=1}{\sum }}\frac{\left({\mathrm{cDNA}\;\mathrm{copy}\;\mathrm{nbr}\;\mathrm{for}\;\mathrm{gene}}_{\left(n\right)}\right)\;\mathrm{in}\;\mathrm{sample}\;A\;\times \;100}{{\mathrm{average}\;\mathrm{cDNA}\;\mathrm{copy}\;\mathrm{nbr}\;\mathrm{for}\;\mathrm{gene}}_{\left(n\right)}\;\mathrm{in}\;\mathrm{all}\;\mathrm{samples}}$$

### 4.9. Data and Statistical Analysis

For each group of the first step, experiments were replicated once with *n* = 3 in each replicate, and the data obtained in each replicate were pooled; therefore, *n* = 6 in each group. For the second step, experiments were performed once with *n* = 3 in each group. For each group, data are expressed as mean ± SD of either the number of copies of the different cDNAs analyzed or the “gene expression”-based virulence index. Statistical significance for within-group comparisons was calculated by a two-way analysis of variance (ANOVA 2) with Tukey’s post hoc test, using XLSTAT Software (version 19.4). The *p* value of 0.05 defined the significance cut-off.

When the *p* value for Factor 1 was below 0.05, meaning that differences between *L. pneumophila* strains were not statistically different, data for *L. pneumophila* strains were pooled together with “*n* = n L. pneumophila Paris + n L. pneumophila Philadelphia + n L. pneumophila Lens.”

For the first step, Factor 1 equals “L. pneumophila strain (Paris, Philadelphia, and Lens)” and Factor 2 equals “culture condition (T′0, T3D-FREE, T3D-WILL, T3D-ACANTH).” For the virulence index, because ANOVA 2 showed no statistical difference between the three strains (*p* = 0.832), values for the three strains were pooled together (*n* = 18).

For the second step, Factor 1 equals “L. pneumophila strain (Paris and Lens)” and Factor 2 equals “culture condition (T′0, T3D-FREE, T6D-FREE, T6D-WILL, T6D-ACANTH).” Strain Philadelphia was not analyzed in this second step because after internalization into both amoebae, it did not grow on BCYE plates in 2 out of 3 samples; because *n* = 1 for this condition, data from strain Philadelphia were not considered for the statistical analysis. Because ANOVA 2 showed no statistical difference between the two other strains (*p* = 0.566), values for both strains were pooled together (*n* = 6).

## Figures and Tables

**Table 1:** The sequences of primer pairs used for qPCR amplification, the slope “a” of the calibration curve: Cq = a × Log[DNA]i + b and the efficiency “E” of the qPCR are given for the targeted cDNAs.

Gene ID	Size (bp)	Forward Primer (5′->3′)	Reverse Primer (5′->3′)	a	E
*ccmF*	149	TGA ATA CAC AGG GCC GTG ATC TGA	ACT GGT TTC TAC TTT CCC TGC CCA	−3.369	1.98
*dotA*	81	CTG AGA TGG ATA GGT GGT AGT C	TCT TAC TCT ACC TTT GGC TTC CTC	−3.473	1.94
*enhC*	438	AAT GCT TTG TAT GCC CTC GG	CAT ATC AGC GCT TTG GCC ATC	−3.401	1.97
*gacA*	120	TTT AAA CGA CGC GTC ACT TCC CAC	TGC AGA TGC TGA AAG TGG TGA GCA	−3.386	1.97
*htpX*	196	ATT GAC TCT CAT GGT TGC CGT GCT	AGC CAT GTA TTC TCT GGT TCG GCT	−3.330	2.00
*icmE*	200	GCT CAA ATC AAA GCT GCT CAG GCA	CCT GCG TTT GCT AAA TCC GCA TCA	−3.331	2.00
*lirR*	124	CCA TGC TTA ATG CTC TCT ACC A	GGG TTG CTC CGC AAT TAA AC	−3.541	1.92
*lvrB*	99	CAT TGG TGT ACT CTC GGT CTT C	AGC ACC ATG CAG AGC ATA C	−3.385	1.97
*lvrE*	128	CCG TAA CAA GTG GGT GAT TCT	CAT TGC CCA ACA AAC CAT AGA C	−3.330	2.00
*rpsL*	132	GAA AGC CTC GTG TGG ACG TA	CAA CCT TAC GCA TAG CTG AGT TA	−3.340	1.99
*tatB*	115	ATT GTG TTT GGG CCA TCA AAG	CAT TGA GTT GTT GCT GCC AAA	−3.484	1.94

## Supplementary Materials

The following are available online at https://www.mdpi.com/2076-0817/9/6/447/s1, Figure S1: Level of *rspL* transcript in the different conditions, expressed as the number of copies in 10^6^ *L. pneumophila* (Lp) ± SD, Figure S2: *ccmF* cDNA copy number measured in 10^6^ *L. pneumophila* for each strain and each culture condition, Figure S3: *gacA* cDNA copy number measured in 10^6^ *L. pneumophila* for each strain and each culture condition, Figure S4: *htpX* cDNA copy number measured in 10^6^ *L. pneumophila* for each strain and each culture condition, Figure S5: *icmE* cDNA copy number measured in 10^6^ *L. pneumophila* for each strain and each culture condition, Figure S6: *lirR* cDNA copy number measured in 10^6^ *L. pneumophila* for each strain and each culture condition, Figure S7: *lvrE* cDNA copy number measured in 10^6^ *L. pneumophila* for each strain and each culture condition, Figure S8: *tatB* cDNA copy number measured in 10^6^ *L. pneumophila* for each strain and each culture condition, Figure S9: *ccmF* and *gacA* cDNA copy number measured in 10^6^ *L. pneumophila* for each strain and each culture condition, Figure S10: *htpX* and *icmE* cDNA copy number measured in 10^6^ *L. pneumophila* for each strain and each culture condition, Figure S11: *lirR* and *lvrE* cDNA copy number measured in 10^6^ *L. pneumophila* for each strain and each culture condition, Figure S12: *tatB* cDNA copy number measured in 10^6^ *L. pneumophila* for each strain and each culture condition, Figure S13: Synoptic diagram of the experiments, Table S1: Fold changes of gene expression after 3-48h exposure of *L. pneumophila* Philadelphia to CuO nanoparticles (CuO-NPs) or synthetic Gray water (Gw).
