# Supplementary material for: Mitigation of Expression of Virulence Genes in Legionella pneumophila Internalized in the Free-Living Amoeba Willaertia magna C2c Maky
Source: Pathogens. 2020 Jun 5;9(6):447. doi: 10.3390/pathogens9060447 (PMC7350332; doi:10.3390/pathogens9060447)
Supplement: Supplementary file 1 [file pathogens-09-00447-s001.pdf]

Short-term exposure: 3-6 h for CuO-NPs and 4 h for Gw		
Gene	**CuO**	**Gw**
cegC1	x10 (high)	no change
csrA	no change	x40
dotA	x20 (high)	*not investigated*
enhC	high	*not investigated*
htpX	low	*not investigated*
icmE	moderate	*not investigated*
icmV	x16 (high)	*not investigated*
icmW	high	*not investigated*
lepA	moderate	*not investigated*
lolA	*not investigated*	x6
pla	*not investigated*	x7
pvcA	high	*not investigated*
sidF	high	x25
umuD	moderate	*not investigated*
**Long-term exposure: 24 h for CuO-NPs and 24-48 h for Gw**		
Gene	**CuO**	**Gw**
ccmF	*not investigated*	x5
cegC1	x10 (high)	no change
dotA	x20 (high)	*not investigated*
enhC	high	*not investigated*
gacA	*not investigated*	x2.5
htpX	low	*not investigated*
icmE	moderte	*not investigated*
lirR	*not investigated*	x15
lvrB	*not investigated*	x65
lvrE	*not investigated*	x15
pvcA	high	*not investigated*
sidF	high	no change
tatB	*not investigated*	x3.5

Table S1: Fold changes of gene expression after 3-48h exposure of *L. pneumophila* Philadelphia to CuO nanoparticles (CuO-NPs) or synthetic Gray water (Gw). Note that changes in sidF were only transient in GW, and that csrA and cegC1 were not modified after exposure to CuO-NPs and Gw, respectively. Adapted from Lu et al., 2013 and Buse et al., 2015 [18-19].

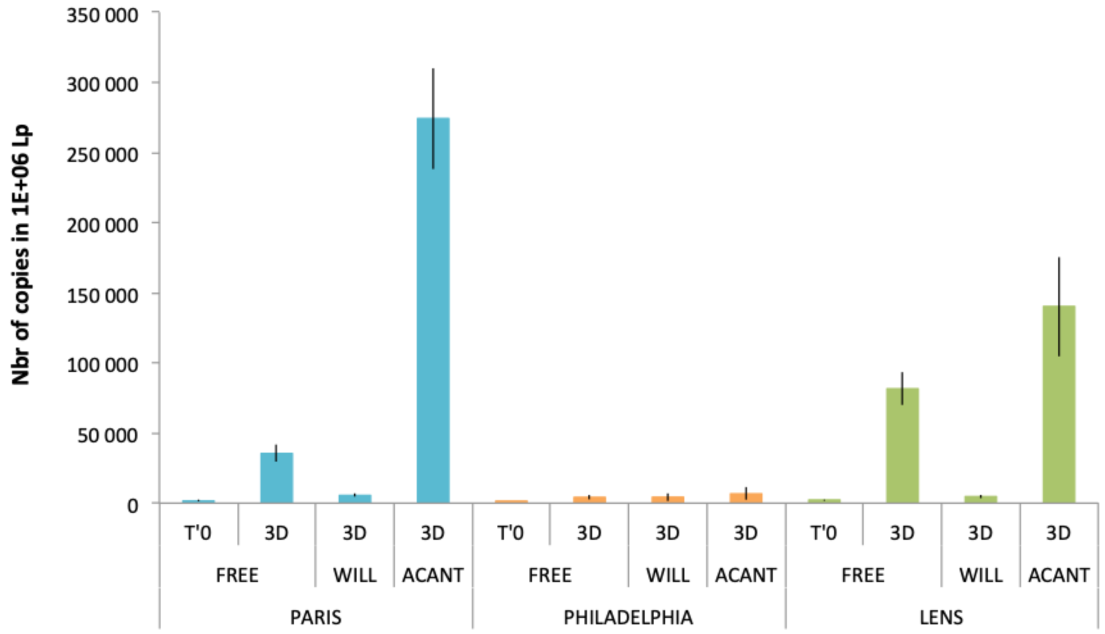

Figure S1: Level of *rspL* transcript in the different conditions, expressed as the number of copies in 10^6 *L. pneumophila* (Lp) ± SD. FREE: *L. pneumophila* strains alone; WILL: *L. pneumophila* strains cocultured with *W. magna* C2c Maky; ACANTH: *L. pneumophila* strains cocultured with *A. castellanii;* T'0: reference transcript level ; 3D: transcript level after 3 days.

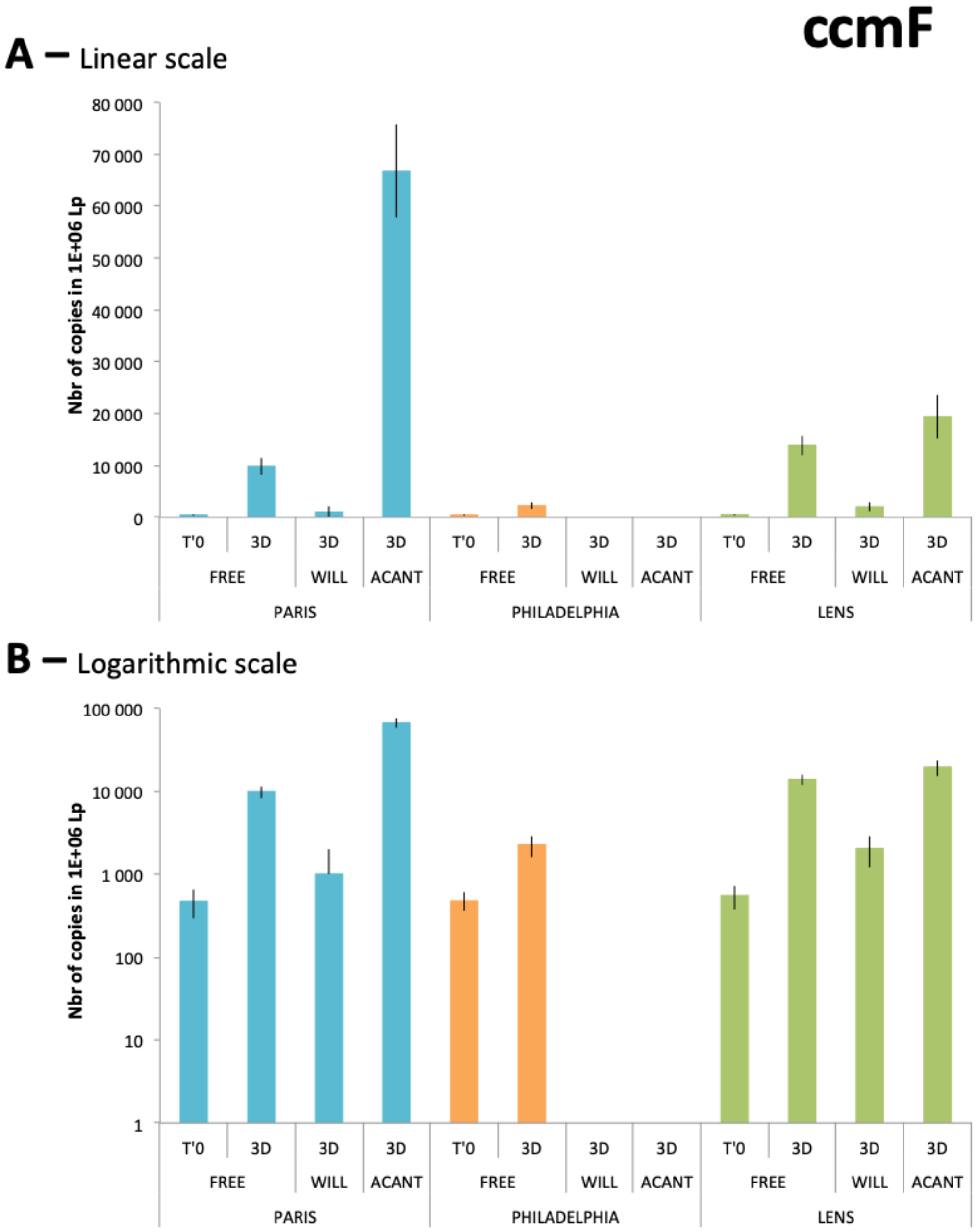

Figure S2: *ccmF* cDNA copy number measured in 10^6 *L. pneumophila* for each strain and each culture condition (see Figure S13 for details). A: linear scale; B: logarithmic scale. Results are expressed as the mean ± SD (n=6, with n=3 in each of the two replicates).

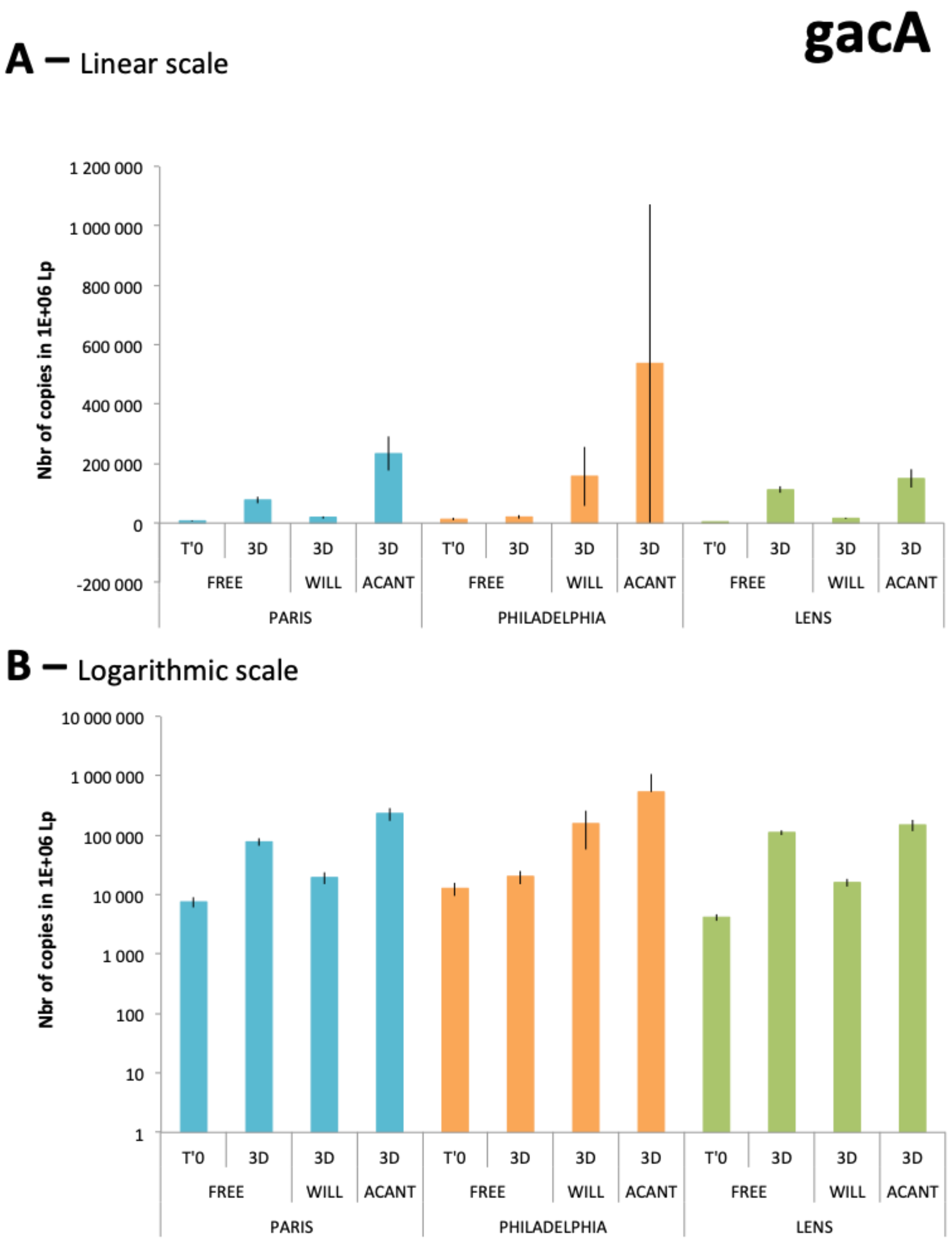

Figure S3: *gacA* cDNA copy number measured in 10^6 *L. pneumophila* for each strain and each culture condition (see Figure S13 for details). A: linear scale; B: logarithmic scale. Results are expressed as the mean ± SD (n=6, with n=3 in each of the two replicates).

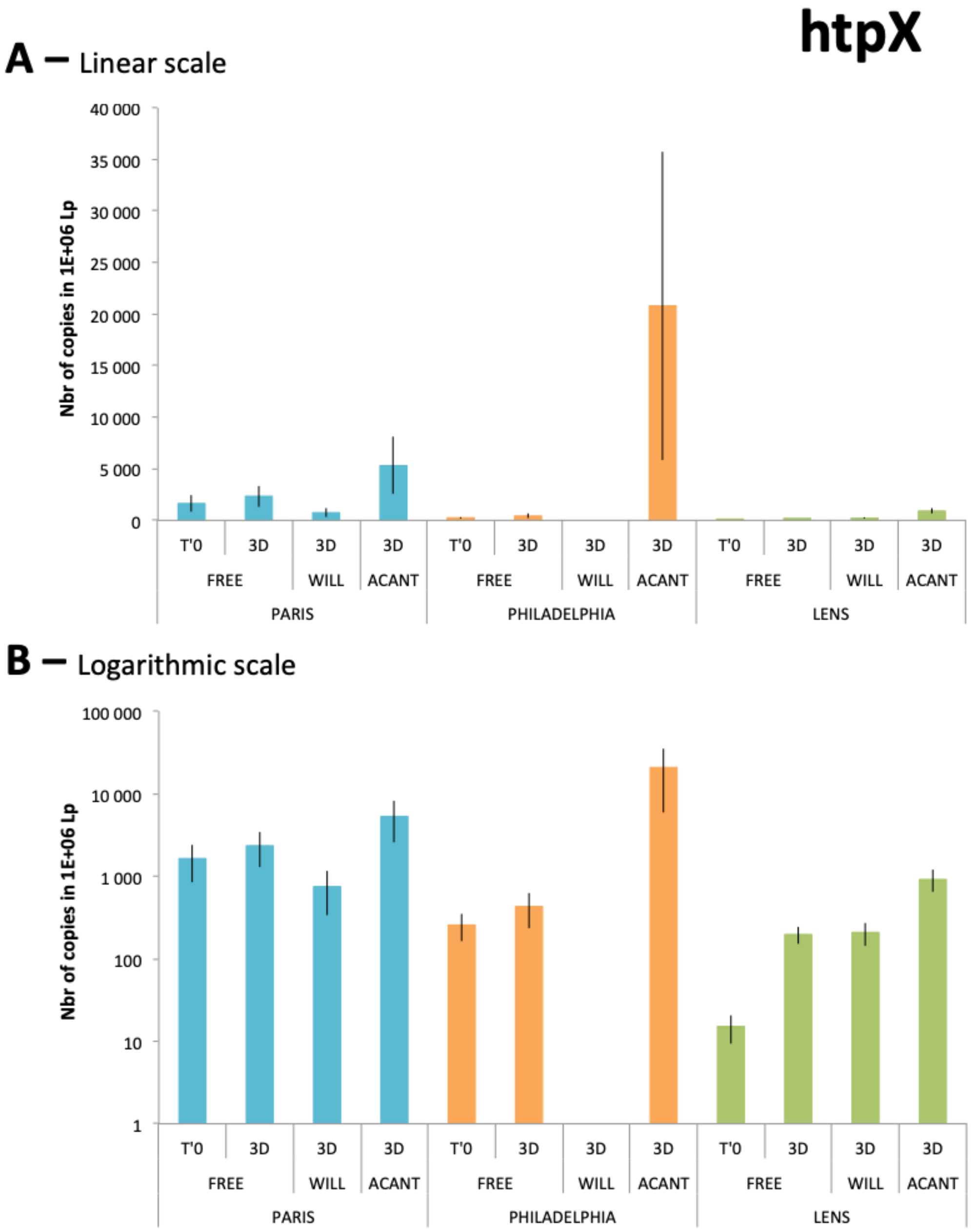

Figure S4: *htpX* cDNA copy number measured in 10^6 *L. pneumophila* for each strain and each culture condition (see Figure S13 for details). A: linear scale; B: logarithmic scale. Results are expressed as the mean ± SD (n=6, with n=3 in each of the two replicates).

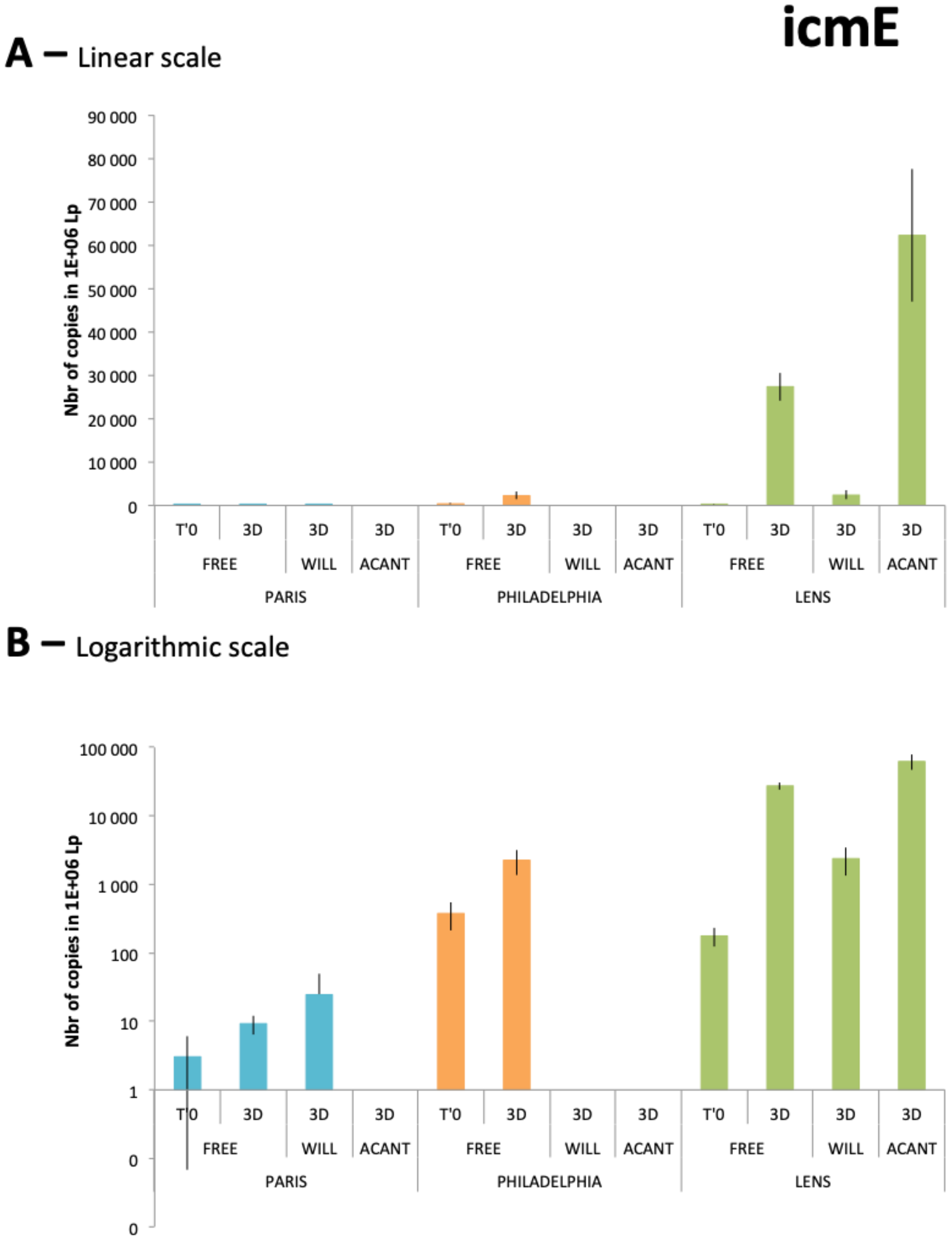

Figure S5: *icmE* cDNA copy number measured in 10^6 *L. pneumophila* for each strain and each culture condition (see Figure S13 for details). A: linear scale; B: logarithmic scale. Results are expressed as the mean ± SD (n=6, with n=3 in each of the two replicates).

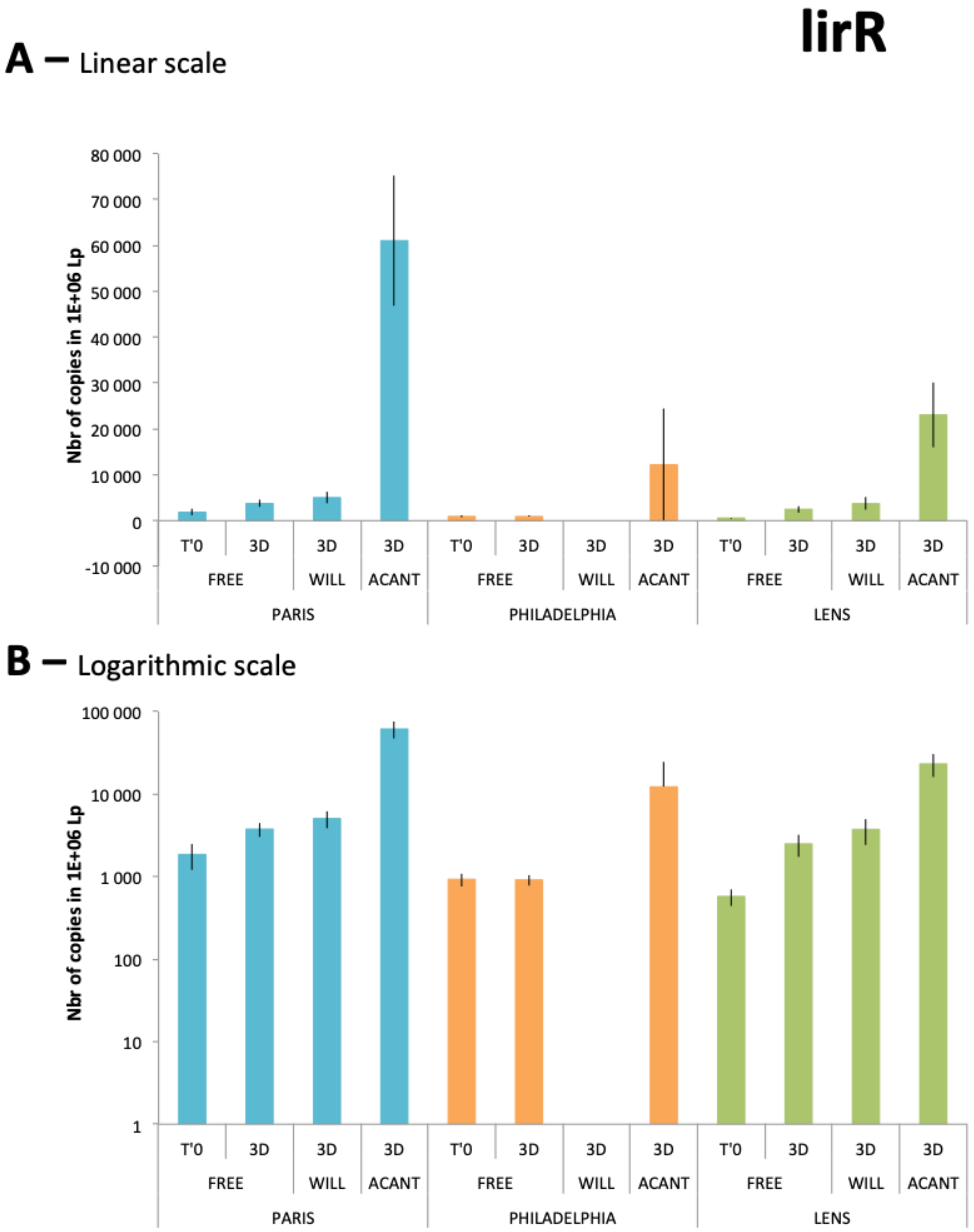

Figure S6: *lirR* cDNA copy number measured in 10^6 *L. pneumophila* for each strain and each culture condition (see Figure S13 for details). A: linear scale; B: logarithmic scale. Results are expressed as the mean ± SD (n=6, with n=3 in each of the two replicates).

lvrE

A – Linear scale

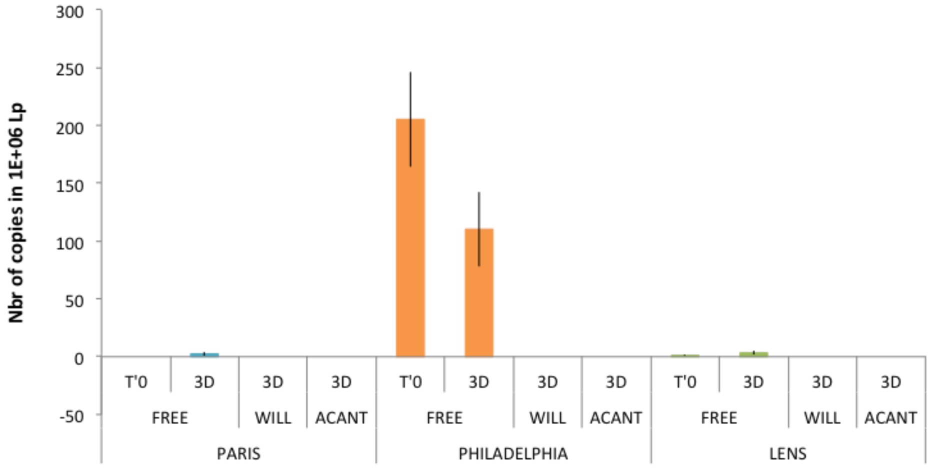

B – Logarithmic scale

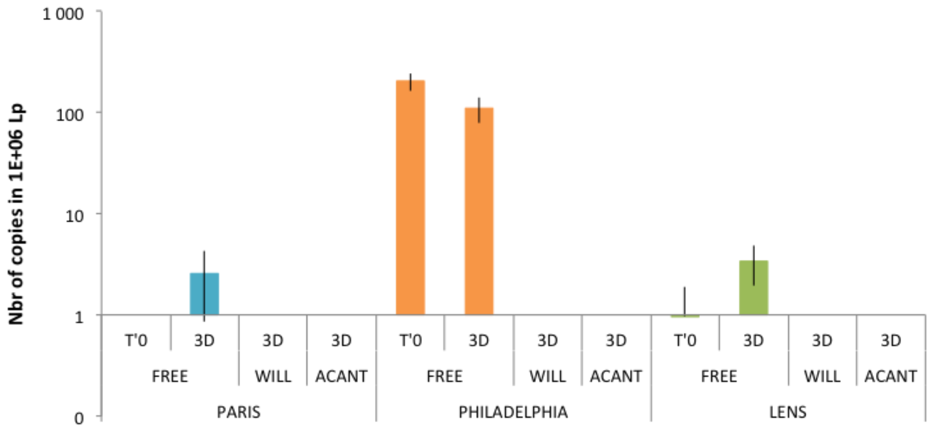

Figure S7: *lvrE* cDNA copy number measured in 10^6 *L. pneumophila* for each strain and each culture condition (see Figure S13 for details). A: linear scale; B: logarithmic scale. Results are expressed as the mean ± SD (n=6, with n=3 in each of the two replicates).

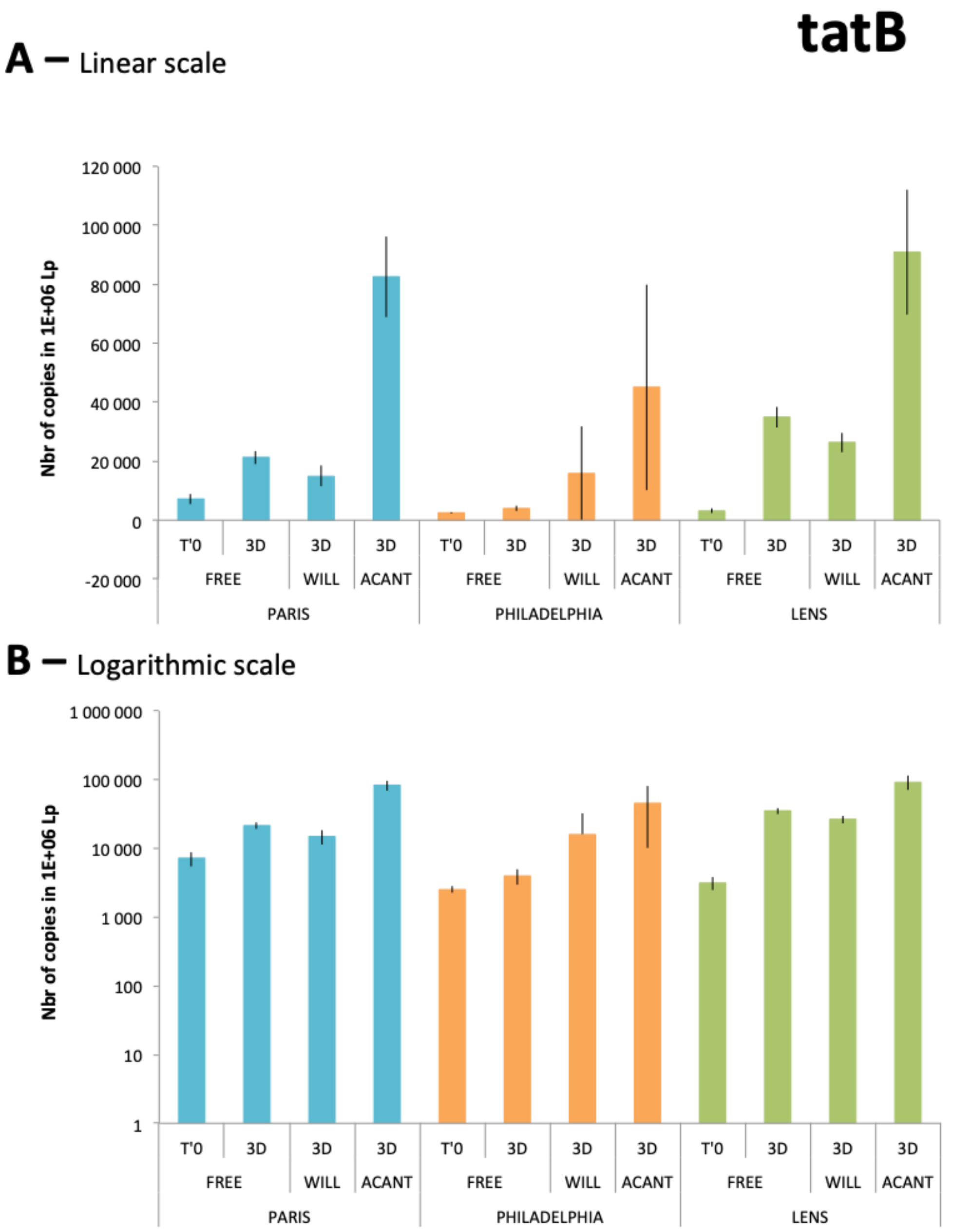

Figure S8: *tatB* cDNA copy number measured in 10^6 *L. pneumophila* for each strain and each culture condition (see Figure S13 for details). A: linear scale; B: logarithmic scale. Results are expressed as the mean ± SD (n=6, with n=3 in each of the two replicates).

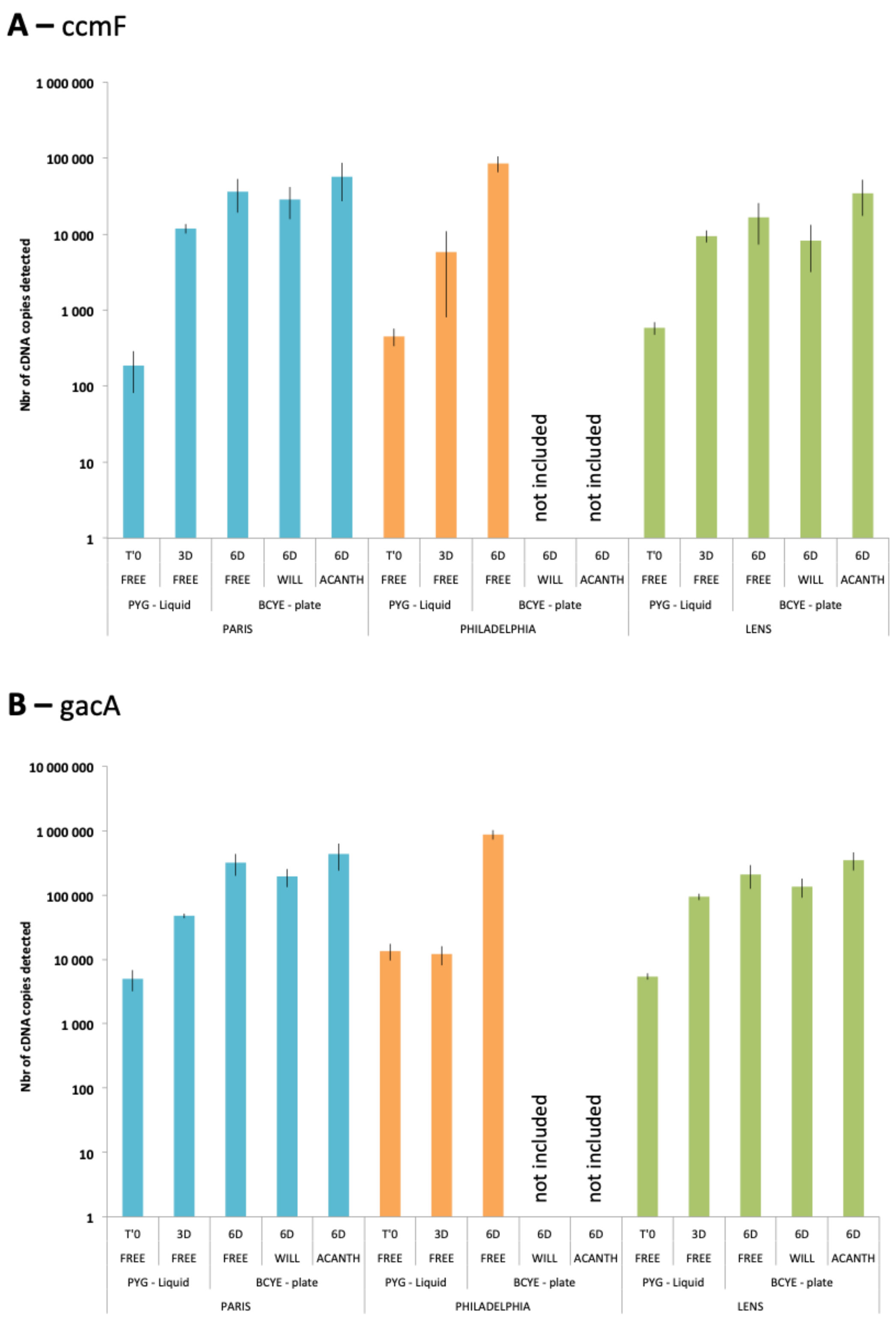

Figure S9: *ccmF* and *gacA* cDNA copy number measured in 10^6 *L. pneumophila* for each strain and each culture condition (see Figure S14 for details). A: *ccmF* gene; B: *gacA* gene. Results are expressed as the mean ± SD (n=3).

A – hptX

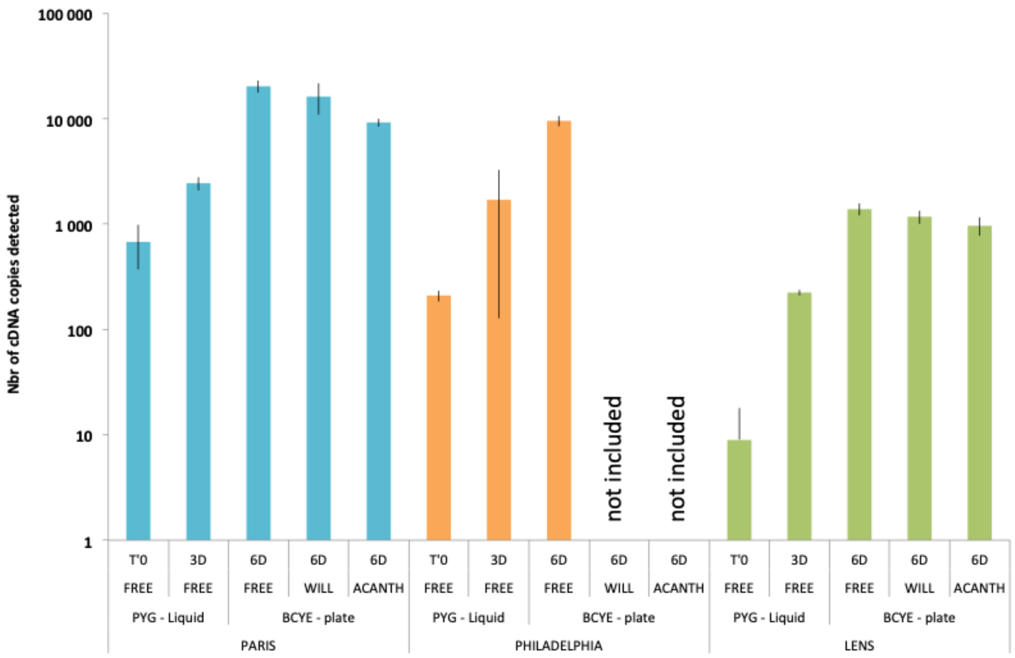

B – icmE

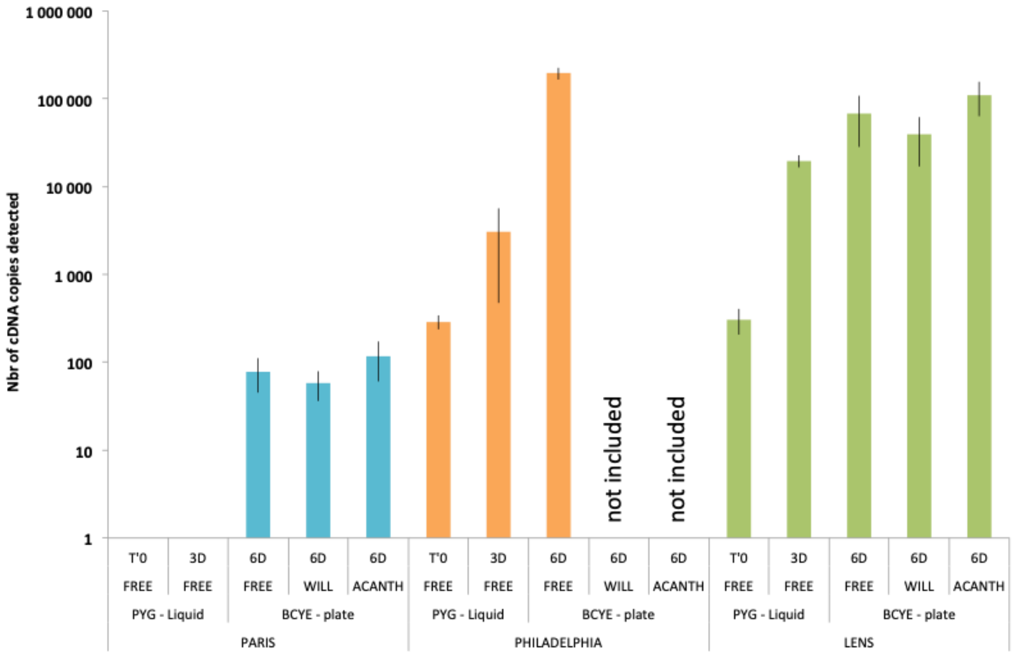

Figure S10: *htpX* and *icmE* cDNA copy number measured in 10^6 *L. pneumophila* for each strain and each culture condition (see Figure S14 for details). A: *hptX* gene; B: *icmE* gene. Results are expressed as the mean ± SD (n=3).

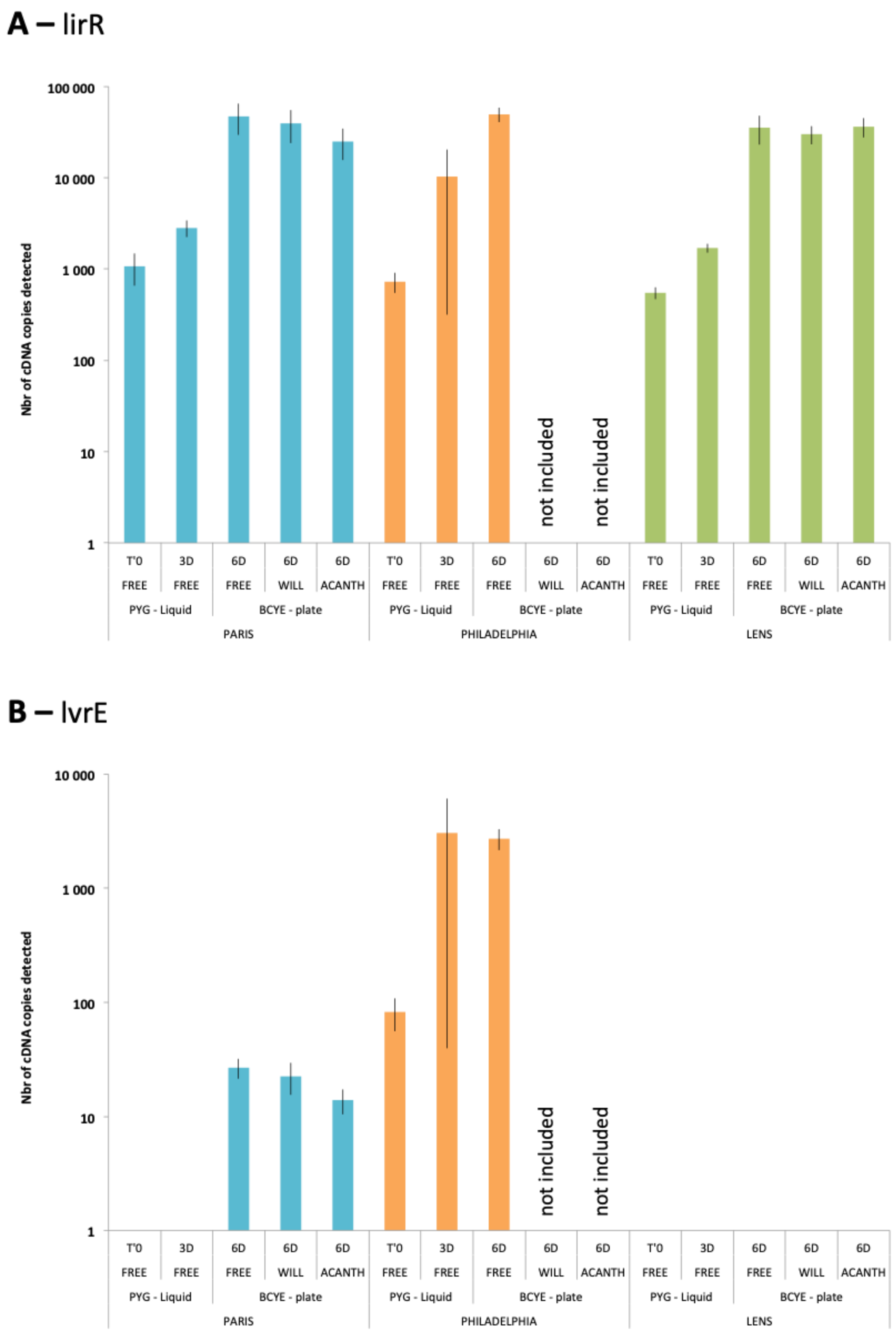

Figure S11: *lirR* and *lvrE* cDNA copy number measured in 10^6 *L. pneumophila* for each strain and each culture condition (see Figure S14 for details). A: *lirR* gene; B: *lvrE* gene. Results are expressed as the mean ± SD (n=3).

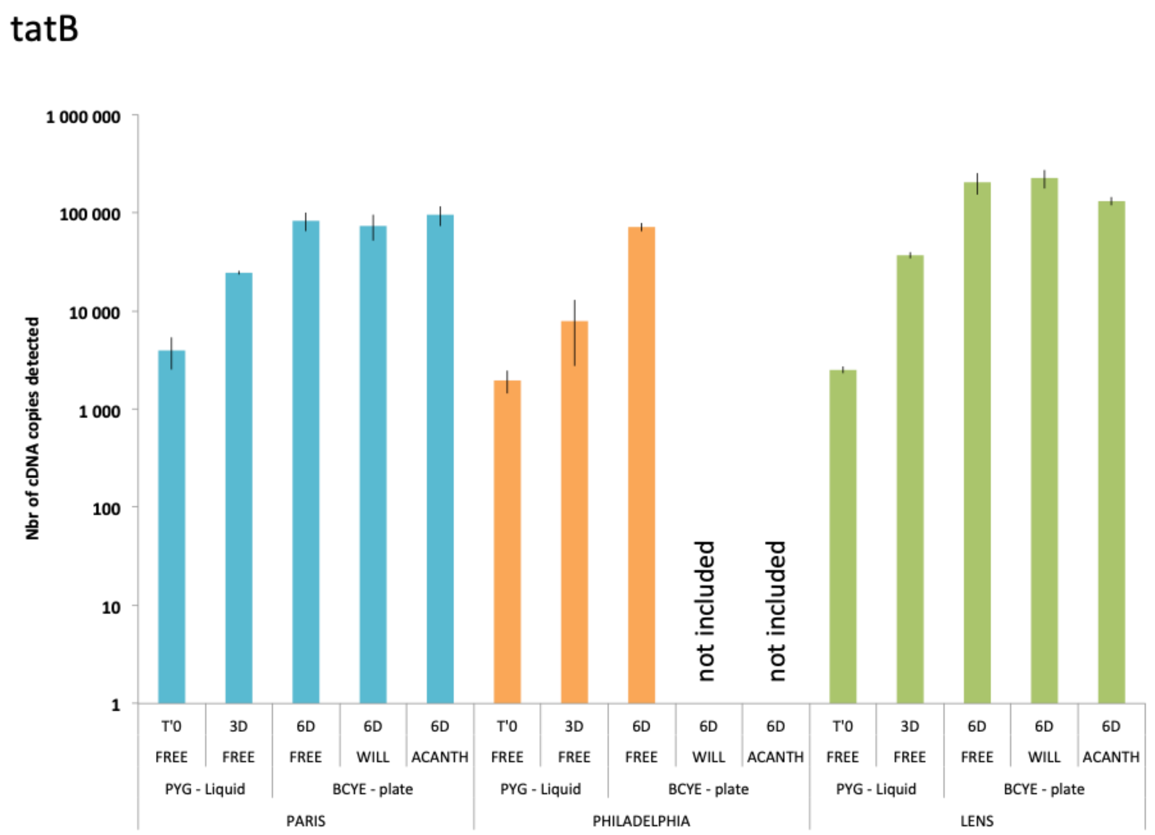

Figure S12: *tatB* cDNA copy number measured in 10^6 *L. pneumophila* for each strain and each culture condition (see Figure S14 for details). Results are expressed as the mean ± SD (n=3).

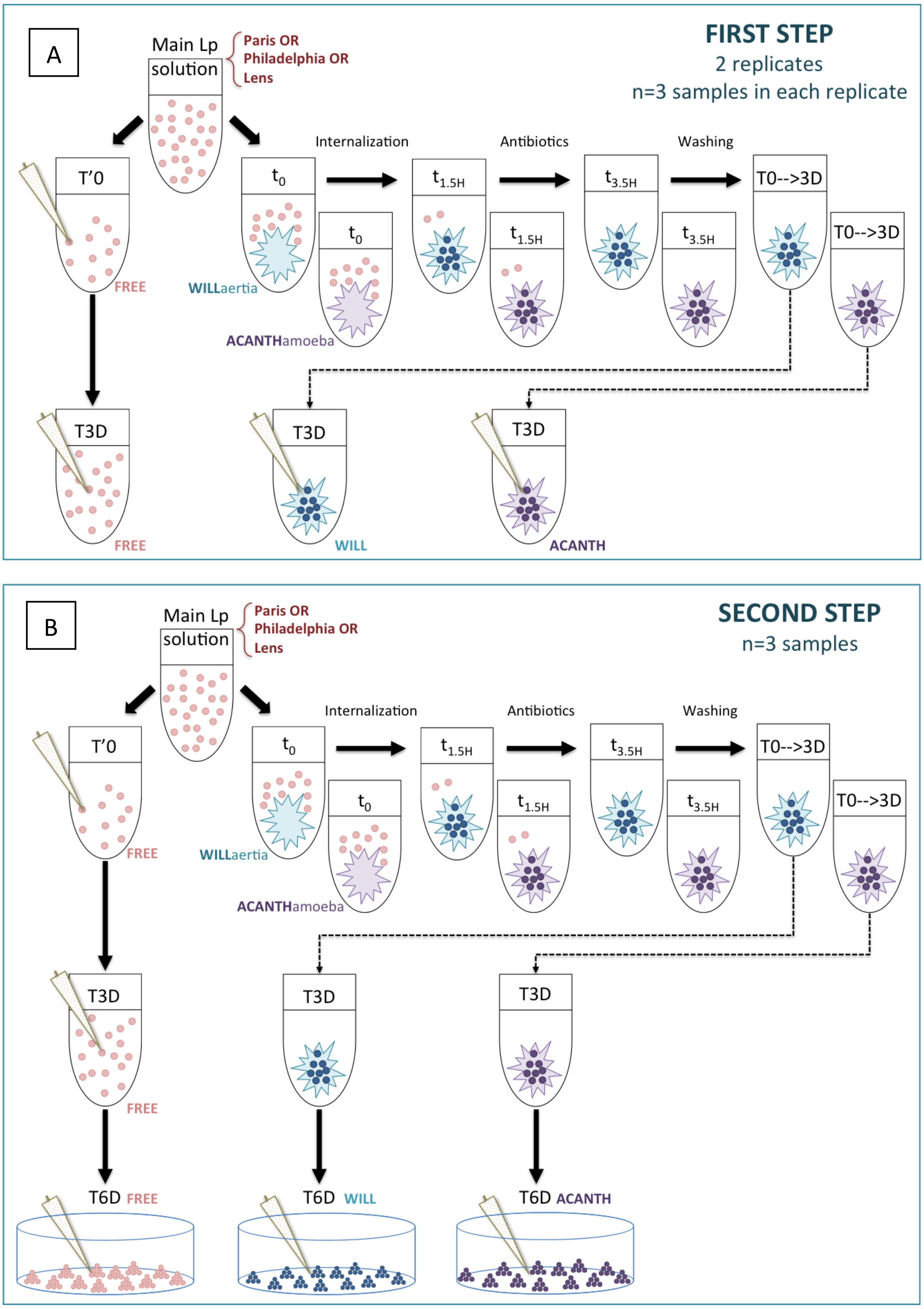

Figure S13: Synoptic diagram of the experiments. A: first step: for the cocultures, after a 1.5h of contact, extracellular *L. pneumophila* were eliminated by antibiotic action for 2h, then the cocultures were rinsed and incubated for 3 days; B: second step: after the 3-day incubation, coculture were spread on BCYE plates and incubated for 3 additional days. Abbreviations: FREE: *L. pneumophila* strains alone; WILL: *W. magna* C2c Maky; ACANTH: *A. castellanii*; T'0: reference sample; T3D: samples collected after 3 days; T6D: samples collected after 6 days, 3 days in liquid medium and 3 days on BCYE plates.
